# Differences in health and well-being of parents of children with disabilities

**DOI:** 10.3402/qhw.v9.24343

**Published:** 2014-04-16

**Authors:** Ulrika Hallberg

**Affiliations:** 1 Nordic School of Public Health (NHV), Gothenburg, Sweden

When a child with a disability is born, reaction of most parents, regardless of gender, span a range of feelings, starting from denial, shock, anger to, hopefully, at last, reconciliation with the circumstances. Different research studies have shown that most of such parents experience increased levels of stress and extended responsibility due to their new situation, and gender differences do determine the experience and reaction to the situation and also the health and well-being of those affected. Women tend to be more affected by stressful life events in general than men, especially when these events affect individuals with whom they have a relationship, such as family members. Women also report a decreased sense of control in stressful situations—they experience more psychological distress and depression and they tend to use more emotion-focused coping-strategies than men such as verbalizing the problems and seeking support from others. Mothers of children with disabilities experience a higher level of stress and depression than fathers. It has been proven that the depressive symptoms of the mothers increase with the age of the child. This is related to the expectance of the child's need for care. A younger child is expected to require more care than an older child, having a disability or not. Mothers of children with disabilities also report a higher rate of marital dissatisfaction while the same does not apply to their fathers. The more severe the mother's subjective perception of the child's disability, the higher the rate of marital discord reported, which is probably because the more severe the child's disability, the more time the mother has to spend with the child, leaving very little time left to be spent with her spouse.

Men are reported to be especially affected by stressful events that are related to work or family finances and appear to be more stoic and less verbal during difficult life events while using more problem-focused coping strategies. In spite of a higher degree of paternal involvement at present than 20 years ago, it has been shown that fathers of children with disabilities take less part in caring for the child than do fathers of children without disabilities. It has been reported that the more the mothers see the fathers as part-taking in the care of the specially abled child, the better the quality of the marital relationship. The fact that fathers report fewer depressive symptoms than do mothers can be explained by their contact with the child being less extensive than that of the mothers. In general, mothers take maximum responsibility in caring for a specially abled child, whereas fathers often tend to focus on employment in order to support the family financially. Fathers report concern for their children regarding the child's self-esteem, progress, learning difficulties, and the risk of being bullied. Furthermore, fathers report stress related to finances, limited social life, and a lack of intimacy with their partners, but, at the same time, they demonstrate a will to protect the mother and they also strive for normalization. The fathers are also concerned about their child's future, the consequences the child's disability might have on the health of the mother and the siblings, their own degree of involvement, and how they might fulfill the financial needs of the family. They also feel a need for spending more time with their partner. Furthermore, the fathers experience stress due to lack of support at their workplace and feelings of worry, guilt, and fear for the child's situation, which can lead to experiences that significantly affect the levels of stress and feelings of life fulfillment. Fathers who report a high degree of marital discord are reported to be more distanced from the child, whereas fathers who see their marriage as good have a closer relationship with the child.

In accordance, mothers’ stress levels are related to the extent of the child's needs and the behavioral problems of the child, whereas fathers’ stress levels are more related to how close the relationship with the child is and the social acceptance of the child. This means that mothers in general benefit more from assistance in caring for the child while fathers benefit more from support in creating a close relationship with the child in order to reduce stress.



*Ulrika Hallberg*
 Nordic School of Public Health (NHV) Gothenburg, Sweden


